# Hypertrophic cardiomyopathy: Mutations to mechanisms to therapies

**DOI:** 10.3389/fphys.2022.975076

**Published:** 2022-09-26

**Authors:** Masataka Kawana, James A. Spudich, Kathleen M. Ruppel

**Affiliations:** ^1^ Department of Biochemistry, Stanford University School of Medicine, Stanford, CA, United States; ^2^ Department of Medicine, Division of Cardiovascular Medicine, Stanford University School of Medicine, Stanford, CA, United States

**Keywords:** myosin, hypertrophic cardiomyopathy, super relaxed state, mavacamten, omecamtiv mercarbil

## Abstract

Hypertrophic cardiomyopathy (HCM) affects more than 1 in 500 people in the general population with an extensive burden of morbidity in the form of arrhythmia, heart failure, and sudden death. More than 25 years since the discovery of the genetic underpinnings of HCM, the field has unveiled significant insights into the primary effects of these genetic mutations, especially for the myosin heavy chain gene, which is one of the most commonly mutated genes. Our group has studied the molecular effects of HCM mutations on human β-cardiac myosin heavy chain using state-of-the-art biochemical and biophysical tools for the past 10 years, combining insights from clinical genetics and structural analyses of cardiac myosin. The overarching hypothesis is that HCM-causing mutations in sarcomere proteins cause hypercontractility at the sarcomere level, and we have shown that an increase in the number of myosin molecules available for interaction with actin is a primary driver. Recently, two pharmaceutical companies have developed small molecule inhibitors of human cardiac myosin to counteract the molecular consequences of HCM pathogenesis. One of these inhibitors (mavacamten) has recently been approved by the FDA after completing a successful phase III trial in HCM patients, and the other (aficamten) is currently being evaluated in a phase III trial. Myosin inhibitors will be the first class of medication used to treat HCM that has both robust clinical trial evidence of efficacy and that targets the fundamental mechanism of HCM pathogenesis. The success of myosin inhibitors in HCM opens the door to finding other new drugs that target the sarcomere directly, as we learn more about the genetics and fundamental mechanisms of this disease.

## Introduction

**FIGURE 1:**
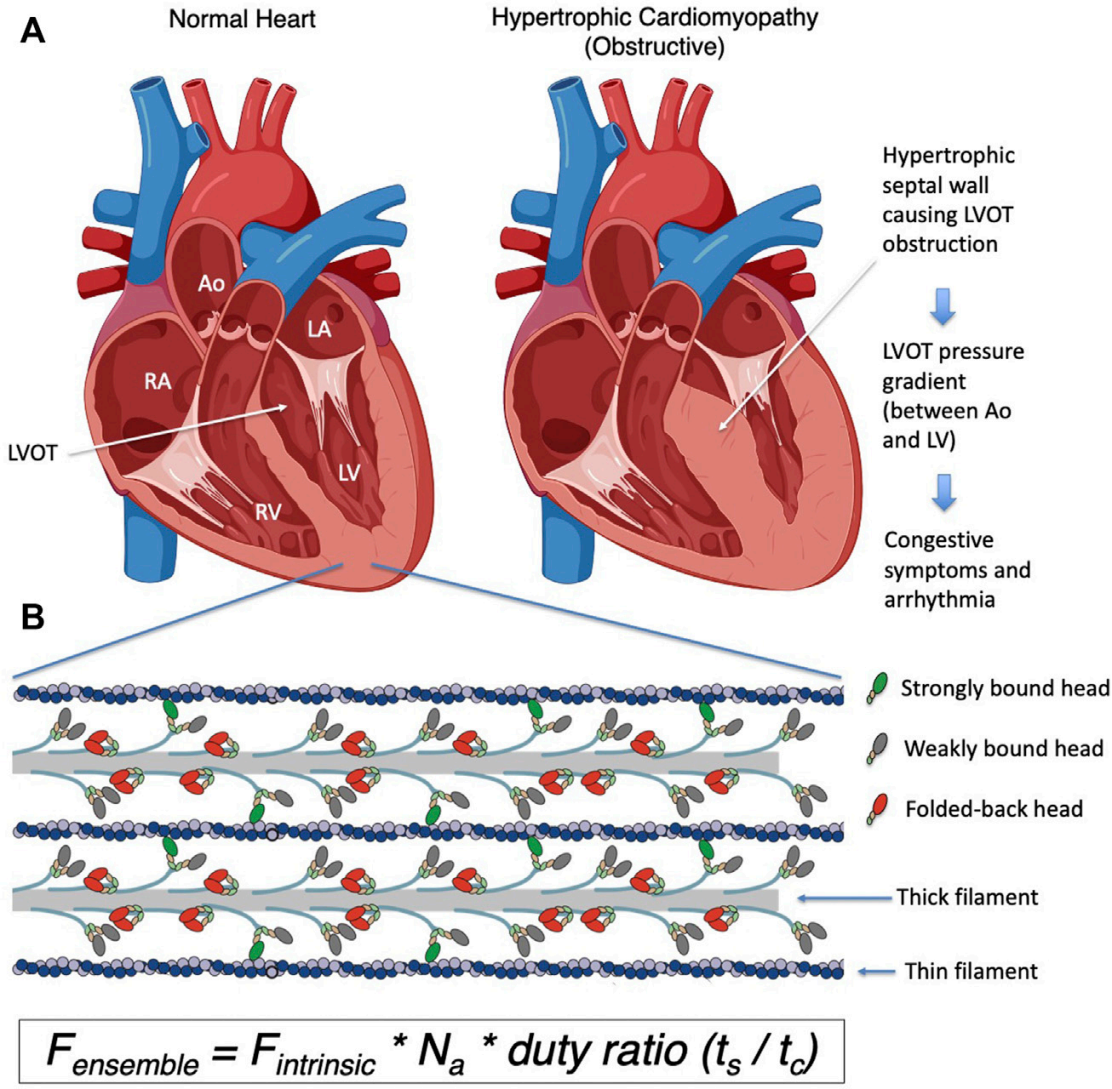
**(A)** Schematic representation of a normal heart and a heart with obstructive hypertrophic cardiomyopathy. The obstruction to blood flow occurs when the septal hypertrophy impedes blood flow from the left ventricular cavity to the aorta *via* the left ventricular outflow tract or LVOT, and a pressure gradient from the left ventricle to the aorta occurs. **(B)** A closeup schematic drawing of a sarcomere, showing the myosin containing thick filaments interdigitating with the actin containing thin filaments. The myosin heads highlighted in red are folded back onto the thick filament and are unavailable to interact with actin. Myosin heads bound to actin are shown in green, and heads that are released from the thick filament but not bound to actin are shown in grey. The number of available myosin heads (N_a_) is the sum of green and grey heads within each sarcomere. The duty ratio is defined as the proportion of the total cycle time (t_c_) an available myosin head spends strongly bound to actin (t_s_). This ratio (t_s_/t_c_) determines the proportion of available heads that are strongly bound to actin in the sarcomere during systole. LA, left atrium; LV, left ventricle; RA, right atrium; RV, right ventricle; Ao, aorta; LVOT, left ventricular outflow tract. Figure 1A was created using BioRender.

Hypertrophic cardiomyopathy (HCM) is the most common form of inherited heart disease, affecting more than 1 in 500 people in the general population (Semsarian et al., 2015). It is characterized by left ventricular hypertrophy (LVH) without an alternative etiology such as aortic stenosis or systemic hypertension, loss of left ventricular cavity size, and is associated with significant comorbidities including heart failure and arrhythmia. The earliest manifestation of the disease is impaired diastolic function and generally hypercontractile left ventricular systolic function by echocardiography. Except for the rare early-onset cases, left ventricular wall thickness is typically normal at first and becomes thicker during adolescence and early adulthood. As wall thickness increases, the size of the LV cavity decreases, and diastolic dysfunction also progresses (Figure 1A). The clinical manifestations are driven by increased pressure in the ventricle from these alterations in the systolic and diastolic function that are fundamentally based on increased myosin-actin crossbridge formation. The increase in left ventricular and atrial pressures leads to elevated pulmonary pressure and congestion which are hallmarks of congestive heart failure, along with an increased risk of both ventricular and atrial arrhythmias. Moreover, patients are at higher risk of sudden cardiac death due to ventricular arrhythmias that are thought to be due to an increased burden of myocardial fibrosis in the hypertrophied ventricle (Seidman and Seidman, 2001).

Until recently, the fundamental mechanism underlying these pathophysiological changes was poorly understood, and hence the available therapies for HCM rely on targeting the secondary physiological changes seen at the organ level. Betablockers and calcium channel blockers are used to suppress the observed hyperdynamic systolic function and arrhythmias. While these agents have been used for decades, there is only anecdotal evidence to support their use in HCM patients and they are often poorly tolerated in young patients. When the LVH primarily involves the septum and obstructs blood flow into the left ventricular outflow tract (LVOT) (Figure 1A), the pressure in the LV further increases, leading to worsening symptoms. Mechanical solutions to this obstruction include either surgically resecting the myocardial tissue that is obstructing the flow (myectomy) or injecting alcohol into the coronary artery that supplies the septum to induce a controlled myocardial infarction to remove excess tissue (alcohol septal ablation). These procedures are invasive and require technical expertise in performing adequate reduction of the obstruction. Hence, a medical therapy that targets the fundamental pathogenesis of HCM and can inhibit and potentially reverse the hypertrophic process, especially hypertrophy that causes the obstruction, has been desired for many decades.

The contractility of the heart is central to cardiovascular physiology and is determined by loading conditions (i.e. preload and afterload) and intrinsic properties of the contractile apparatus of the cardiac myocyte. The sarcomere–comprised of interdigitating myosin-containing thick filaments and actin-containing thin filaments–is the fundamental unit of the contractile apparatus (Figure 1B). Myosin is the motor protein that uses the energy produced by hydrolysis of ATP to undergo a power stroke when bound to actin to create force and sarcomere shortening, which is the basis of cardiac contraction. Other proteins in the sarcomere, such as cardiac myosin binding protein C (MyBP-C) and titin, modulate the effect and efficiency of this actin-myosin interaction in response to physiological stimulation and pharmacological intervention. As discussed below, many genetic variants in sarcomere proteins are known to cause hypertrophic and dilated cardiomyopathies, suggesting that subtle changes in sarcomere function can lead to a significant change in myocardial morphology and function. Thus, understanding the mechanism of disease pathogenesis requires a molecular analysis of this actin-myosin interaction.

In this review, we summarize our current understanding of the molecular basis of contractility, our strategy for assessing molecular determinants of contractility, progress in understanding the effect of mutations in cardiac myosin that cause hypertrophic cardiomyopathy, and the recent development of novel sarcomere modulators. Given the space limitation and the importance of using human proteins (described below) in assessing the mechanism of HCM, this review focuses primarily on studies that utilized a reconstituted system of expressed and purified human proteins, as well as some studies of human-induced pluripotent stem cell-derived cardiomyocytes (hiPSC-CM).

## Understanding the ensemble force of the sarcomere

### Biochemical and biophysical determinants of myosin function

We begin by defining the molecular determinants of contractility. By the mid-20th century, biochemists and physiologists had determined the basic components of the contractile elements of muscle to be myosin and actin (Huxley and Hanson, 1959), as well as regulatory units of actomyosin crossbridge interactions including tropomyosin and the troponin complex. Myosin is a hexamer comprised of two heavy chains and two pairs of light chains. The heavy chains form a coiled-coil rod to form an elongated tail, and globular heads which interact with actin. Myosin and myosin binding protein C (MyBP-C) comprise the major components of the thick filament, whereas actin, troponin and tropomyosin form regulated thin filaments (RTF) (Bailey et al., 1946). Hugh Huxley provided compelling evidence for a sliding filament model of muscle contraction based on observations of thin and thick filaments by electron microscopy and on X-ray diffraction patterns in the 1950s, and further proposed a swinging cross-bridge hypothesis of muscle contraction in 1969 (Huxley, 1969), which has been supported over the years as we have learned details of how myosin works as a molecular motor.

**FIGURE 2:**
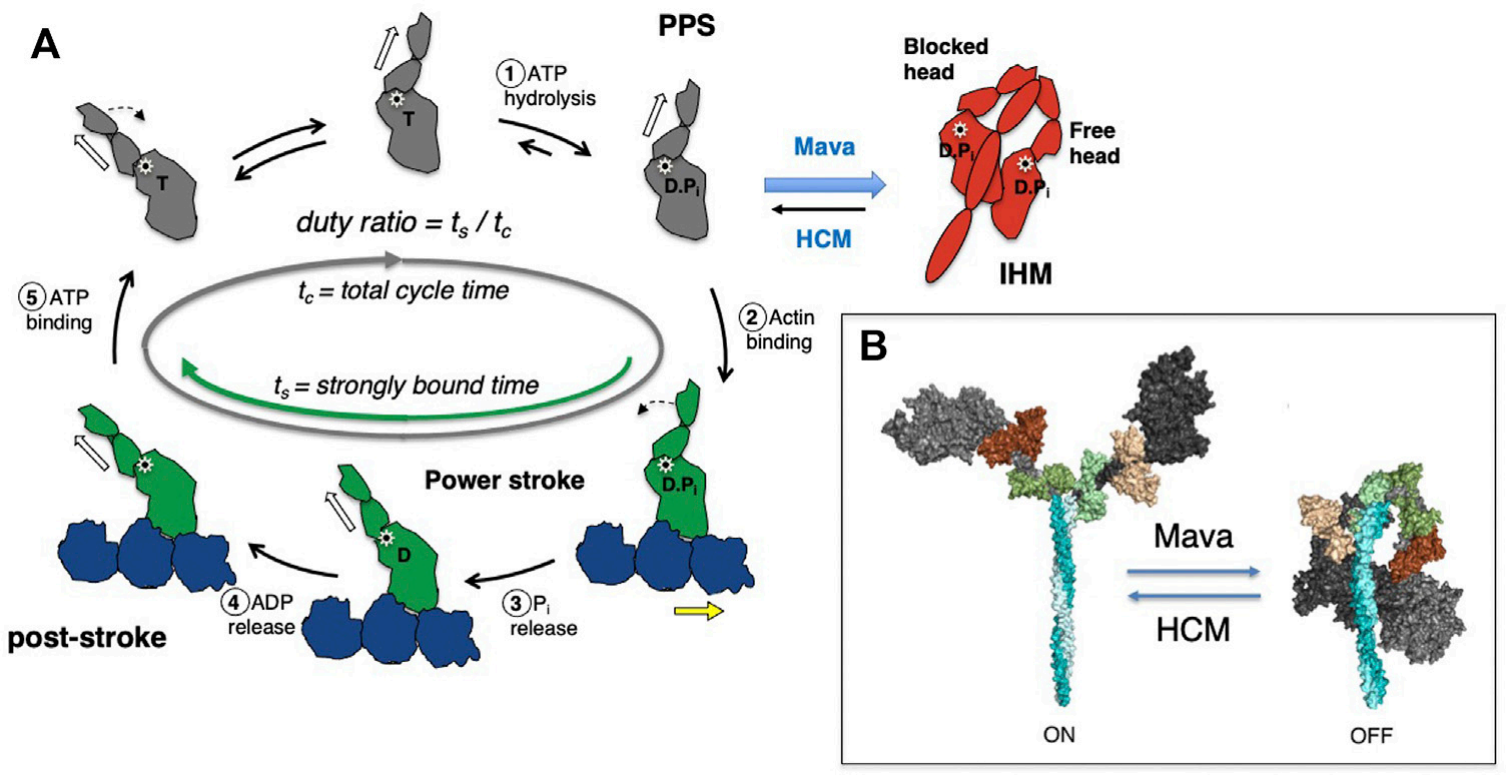
**(A)** Chemomechanical cycle of myosin interacting with actin. Only one head (S1 motor) is being depicted in the cycle for clarity. *1*) S1 hydrolyzes ATP to ADP and phosphate (Pi), which remain bound in the active site, stabilizing the pre-powerstroke state (PPS, white arrow is tilted to the right). *2*) The PPS S1-ADP-Pi binds strongly (grey to green) to actin (blue). *3*) Pi is released while S1 is bound to actin and the lever arm swings to the left (black dashed arrow) about a fulcrum point (black star), moving the actin filament to the right (yellow arrow) with respect to the myosin thick filament (power stroke). *4*) ADP release allows a further small stroke to the poststroke position (white arrow to the left) and frees the active site for binding of ATP. *5*) ATP binds and weakens the interaction of S1 and actin, releasing S1 from the actin filament. S1 undergoes a recovery stroke to the PPS state (dotted arrow; white arrow moves from the left to the right). S1 in the pre-powerstroke configuration can be pulled into the folded IHM state (red). Mavacamten (Mava) affects the equilibrium of these heads as shown, and stabilizes the IHM form, whereas HCM mutations destabilize the IHM in many cases and shift more myosin into the cycle. In the cycle, weakly bound and detached S1 motors are shown in gray, and strongly bound motors are shown in green. **(B)** Structural homology models of cardiac myosin showing the effect of HCM mutations shifting the equilibrium toward the open state (ON), and mavacamten treatment shifting the equilibrium toward the folded state (OFF).

Myosin is the first protein to be identified as a mechanoenzyme–an enzyme that uses the chemical energy from hydrolysis of ATP and converts it to mechanical movement–in this case the displacement of actin filaments within the sarcomere (Figure 2A). Upon ATP binding, myosin detaches from actin and hydrolyzes the bound ATP to ADP*Pi, which remain bound to the active site. With this binding and hydrolysis, the motor exhibits significant structural changes from a post-stroke to a pre-powerstroke (PPS) state. Myosin in the PPS state then binds to actin, which triggers release of the Pi associated with a major part of the power stroke. ADP is then released, accompanied by a small further stroke to complete the power stroke. This post-stroke state myosin is released from actin when ATP binds to the nucleotide binding pocket.

We focus experimentally on key parameters of the above described chemomechanical cycle as follows: First, in cardiac myocytes where ATP concentration is saturating and the myosin motor has a low duty ratio (described below), the rate-limiting step of the whole cycle is the phosphate release rate (k_cat_), and the inverse of k_cat_ equals the total cycle time (t_c_) of the whole reaction cycle. Within this cycle, particular interest is paid to how long the myosin stays strongly bound to actin (the duration is termed the strongly bound state time, or t_s_). The ratio of t_s_ and t_c_ (= t_s_/t_c_) is termed the “duty ratio” of the actin-myosin interaction in the sarcomere (Figure 2A). N_a_ is the number of myosin heads that are accessible to interact with actin, and this is related to the conformation of myosin in the thick filament (Figure 2B). The power stroke produced by the myosin produces force against the actin filament and displaces the actin filament. The distance by which actin is displaced by the myosin power stroke is termed stroke size (d), and the force generated by an individual myosin power stroke is termed intrinsic force (F_intrinsic_). These parameters are related to the physical properties of the myosin motor (Uyeda et al., 1990). As every myosin head acts as an independent force generator, we define the net contractile force of the sarcomere, or ensemble force (F_ensemble_, Figure 1B), using the above fundamental parameters of actin-myosin crossbridge formation as follows (Spudich, 2014):

$$\begin{array}{l} F_{\text{ensemble}}=F_{\text{intrinsic}}\;*\;N_{a}\;*\;\mathrm{duty}\;\text{ratio}\\ =F_{\text{intrinsic}}\;*\;N_{a}\;*\;t_{S}/t_{C} \end{array}$$

**FIGURE 3:**
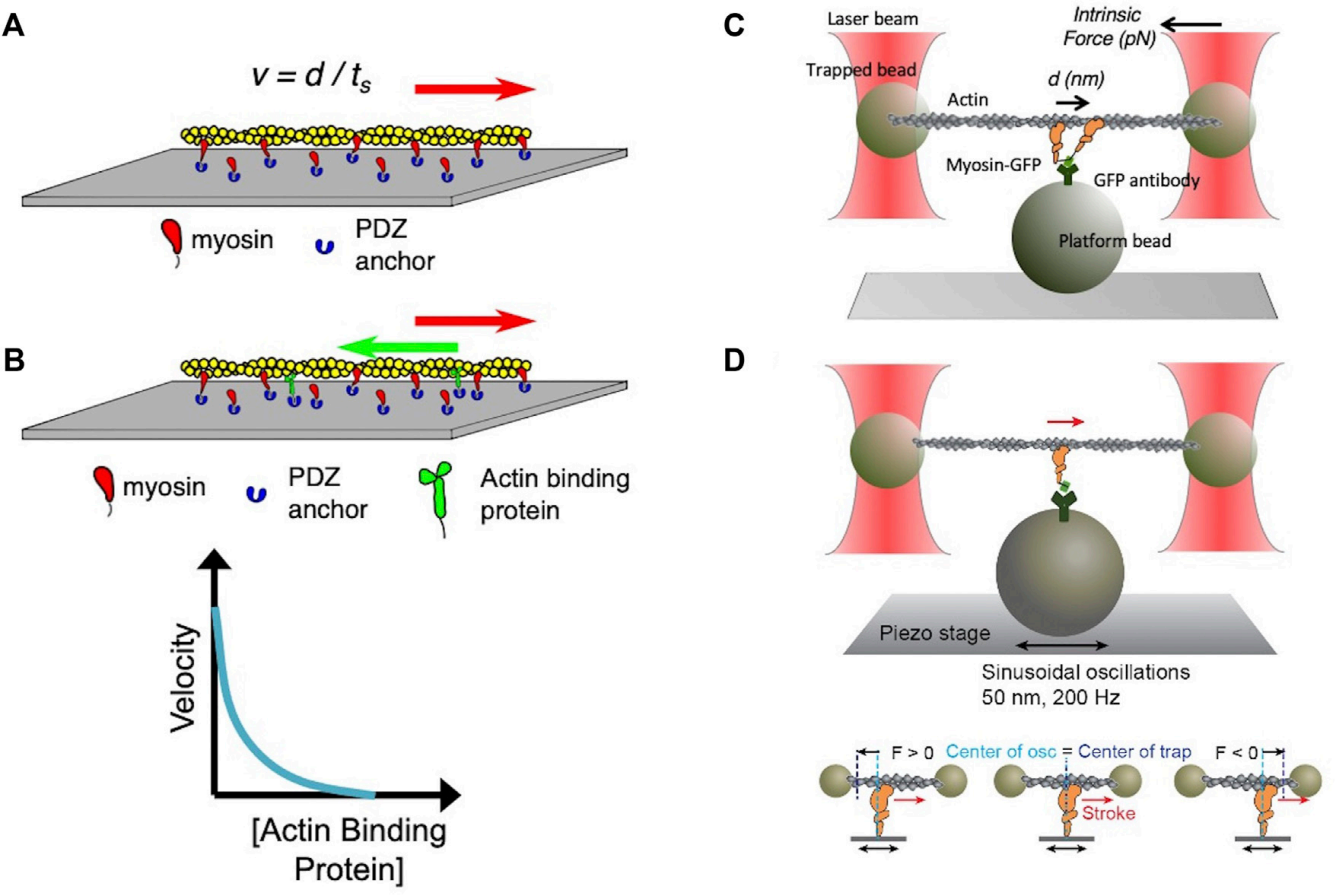
**(A)** Schematic of an *in vitro* motility assay. Myosin is anchored with its C-terminus bound *via* an anchoring peptide to a PDZ protein on a glass surface. Fluorescently labeled actin is added, and in the presence of ATP the actin filament is propelled by the myosin head powerstrokes. The unloaded gliding velocity is proportional to the powerstroke (d) divided by the strongly bound state time (t_s_). **(B)** Schematic of a loaded *in vitro* motility assay. Myosin and an actin-binding protein (such as utrophin) are anchored on the surface, and fluorescent actin filaments are added in the presence of ATP. The actin-binding protein impedes (puts a load on) the actin filament movement. The velocity of the gliding filament is plotted against the concentration of the actin-binding protein to obtain a force-velocity curve. **(C)** Schematic of the unloaded single molecule optical trap assay. Two beads coated with neutravidin are trapped by an infrared laser beam, and a biotinylated actin filament is attached at its ends to the two optically trapped beads to form an actin dumbbell. The position of each of the trapped beads is determined accurately by two position-sensitive detectors (PSD) located in the optical path above the trapped beads. Myosin is attached to the platform bead at a very low concentration to ensure only one myosin is attached to the platform bead to measure single molecule behavior. As the myosin interacts with the actin dumbbell, the myosin produces a stroke (the single head is seen to stroke to the right). At low trap force compared to the force produced by the myosin, the distance of the stroke (d) is measured by the displacement of the trapped bead’s positions. Intrinsic force is measured by applying an instantaneous counter force by increasing the trap strength to match the myosin force, bringing the beads to the original position. The matching trap force can be read out. **(D)** Schematic of the harmonic force spectroscopy optical trap. Oscillations of the piezo stage on which myosin (orange) is attached in the three-bead optical trap system (top) apply a sinusoidal load (force) to myosin upon attachment to actin (bottom). The time myosin is bound to actin (t_s_) is recorded and analyzed against the applied load on the myosin molecule to obtain detachment rates and force sensitivity. Figures adapted from Aksel et al. (Aksel et al., 2015) and Liu et al. (Liu et al., 2018).

Various experimental methods have been developed to study each parameter in detail. First, biochemical and structural analysis of the actin-myosin ATPase cycle has been vital to our understanding of sarcomere function (De La Cruz and Ostap, 2004; Sweeney and Houdusse, 2010; Walklate et al., 2016). Measurements of the rate and equilibrium constants of each step in the cycle, including actin-myosin binding and dissociation, ATP binding, ATP hydrolysis, phosphate release, and ADP release and binding have been extensively performed (*see* review by De La Cruz and Ostap (De La Cruz and Michael Ostap, 2009)). As the rate-limiting step of the cardiac myosin cycle is the release of phosphate from the actin-myosin-ADP*Pi complex, its ATPase activity is determined by an actin-activated ATPase assay measuring the rate of phosphate release (De La Cruz and Michael Ostap, 2009; Sommese et al., 2013a; Nag et al., 2015; Adhikari et al., 2016; Kawana et al., 2017). To interrogate the biophysical aspects of the actin-myosin interaction, *in vitro* motility assays were developed by Kron and Spudich (Kron and Spudich, 1986) and Yanagida et al. (Yanagida et al., 1984) in the 1980s. In the Kron and Spudich assay fluorescently-labeled actin filaments are observed sliding over a glass surface coated with myosin (Figure 3A), enabling the visualization of the displacement of actin filaments by the motor protein under a microscope. The velocity of a sliding actin filament is proportional to the distance moved divided by time (Uyeda et al., 1990; Uyeda et al., 1996); in this case the myosin stroke size divided by the time that myosin spends strongly bound to actin. Hence, v ∝ d/t_s_. *In vitro* motility assays have been used to characterize the function of different isoforms of myosin, including the cardiac isoforms (UP et al., 2004; Debold et al., 2007; Lowey et al., 2008). When myosin is the only protein on the surface capable of interacting with actin and bovine serum albumin is used to block non-specific interactions between the actin filament and the surface, the assay is assumed to represent an unloaded state of actomyosin interaction. As muscle is always operating under some load *in vivo*, modification of the *in vitro* motility assay was done to simulate more physiological conditions by adding an actin-binding protein on the surface (Greenberg et al., 2010; Greenberg and Moore, 2010; Aksel et al., 2015) to create a “loaded *in vitro* motility assay” (Figure 3B). Further spatial optimization of the *in vitro* motility assay has been carried out using a DNA nanotube scaffold that allows precise spacing of a defined number of myosin motors on a track along which actin filaments can be propelled, more closely mimicking the natural organization of the myosin and actin in the muscle sarcomere (Hariadi et al., 2015).

In the 1990s, Finer et al. (Finer et al., 1994) built a dual-beam laser trap for single-molecule analysis that allowed them to measure fundamental aspects of the actomyosin interaction including the distance by which myosin moves actin during a single ATPase cycle (stroke size d ≈ 10 nm) and the intrinsic force a single myosin exerts on an actin filament (F_intrinsic_ ≈ 5 pN) (Figure 3C). In the past several decades, the laser trap system has been used by many other laboratories, and it has undergone continuous upgrading to allow increasingly precise measurements of d, F_intrinsic_ and t_s_ (Sung et al., 2010). Furthermore, the use of dual-beam laser traps with either a high-speed feedback system or harmonic force spectroscopy (HFS) (Veigel et al., 2003; Veigel et al., 2005; Sung et al., 2015) allowed the measurement of load-dependent changes in myosin function at the single molecule level. In HFS, the durations of binding events between a single myosin and an actin filament under different load forces are measured at ATP concentrations (2 mM) approaching physiological (Figure 3D). The sample stage oscillates sinusoidally so that by the randomness of where myosin initially attaches to actin, a range of mean forces are automatically applied over the course of many binding events (Sung et al., 2015; Vander Roest et al., 2021). This technique has been used to quantify changes in the load-dependent detachment of myosin from actin filaments.

Both the *in vitro* motility assay and the single molecule assay have been used to study a wide variety of muscle and non-muscle myosin motors. However, *in vitro* studies of mutated forms of human striated muscle myosins were limited to biopsy samples due to the difficulty in expressing functional recombinant cardiac or skeletal muscle myosins. With the demonstration that striated muscle motors expressed in a mouse myoblast cell line containing muscle-specific chaperone proteins are fully functional, a new era was ushered in in which recombinant human cardiac myosin (Srikakulam and Winkelmann, 2004; Liu et al., 2008; Resnicow et al., 2010) can be highly purified and studied using these and other assays, as detailed below.

### Myosin conformation determines the number of myosin heads functionally accessible to interact with actin in the sarcomere

The above-described enzymatic and biophysical assays have been the main tools used to study the function of myosin as a motor protein. However, while we studied the effects of HCM-causing mutations on β-cardiac myosin motor function as described below, it became apparent that in order to have a full understanding of sarcomere function using the F_ensemble_ calculation, we needed to understand what determines N_a_. It was already well understood that the troponin-tropomyosin containing thin filament regulates the interaction between myosin heads and actin by blocking the ability of myosin to bind to the thin filament when Ca^2+^ concentrations are low (i.e. diastole, *see* part 3 below). Over the last several years, it has become increasingly clear that the actomyosin interaction is also regulated at the level of the thick filament.

Structural evidence for thick filament-mediated regulation of N_a_ was first provided by Wendt and others who determined a cryo-electron microscopic structure of unphosphorylated smooth muscle myosin (Wendt et al., 1999) in which the motor domains are folded-back asymmetrically onto the proximal portion of the coiled-coil tail. This folded-back state of myosin, later termed the “interacting heads motif” or IHM, was postulated to represent a sequestered state of smooth muscle myosin in which the motor domains were positioned such that one or both heads were unavailable to interact with actin. Subsequently, similar folded structures were reported for other myosins including skeletal (Woodhead et al., 2005) and cardiac myosin (Zoghbi et al., 2008), and an electron microscopy study showed that the proximal S2 region of the myosin tail is involved in stabilizing the IHM structure (Lee et al., 2018). When myosin forms the IHM, these myosin heads appear to be sequestered onto the thick filament backbone (Craig and Woodhead, 2006; Brunello et al., 2020), further making them functionally unavailable for actin interaction and thus reducing N_a_ (red motors in Figures 1B, 2B).

This potential mechanism for switching on/off myosin in muscle and non-muscle cells by IHM formation has been observed in a variety of species (Craig and Woodhead, 2006; Jung et al., 2008; Lowey and Trybus, 2010) and the potential effects of HCM-causing mutations on IHM formation were proposed by Moore, Leinwand and Warshaw (Moore et al., 2012). Given that the folded state of myosin was thought to conserve energy utilization (discussed in more detail below), the dysregulation of structural stability in cardiac myosin was thought to be an attractive explanation for altered energetics seen in HCM patients (Ashrafian et al., 2003; Moore et al., 2012; van der Velden et al., 2018). Our laboratory was the first to test this hypothesis using purified recombinant human β-cardiac myosin containing HCM mutations. It was noted that a relatively flat surface of the myosin motor domain, termed the “myosin mesa,” contained highly conserved residues from mouse to human and was enriched for residues mutated in patients with HCM (Spudich, 2015; Homburger et al., 2016). This myosin mesa appeared to work as a docking platform for another protein to bind, thereby sequestering these heads into an inactive state (Spudich, 2015; Homburger et al., 2016). Nag et al. (Nag et al., 2017) tested whether the S2 tail or MyBP-C might act as this sequestering protein. They created recombinant human cardiac HMM constructs with either 25 heptad repeats of the proximal S2 tail (long tail - able to form a structure in which the heads fold back against the tail) or two heptad repeats (short tail - head cannot stably fold back) and showed that the presence of the long tail, but not the short tail, inhibits the actin-activated ATPase activity of the myosin (Nag et al., 2017). They further showed that this inhibition is reduced by phosphorylating the myosin regulatory light chain. They then assessed the binding affinity of the head and tail domain using microscale thermophoresis (MST), which follows the diffusion of a fluorescent probe along a temperature gradient and is used to measure bi-molecular interactions (Wienken et al., 2010; Ponnam and Kampourakis, 2022). This technique has been used to measure binding affinity of protein-protein or protein-small molecule interactions over a broad range of affinities, including K_d_ values in the double-digit μM range, suitable for characterization of protein interactions in the sarcomere (Nag et al., 2017; Adhikari et al., 2019). Similar to previous analytical centrifugation studies that assayed the binding between MyBP-C and S2 or the regulatory light chain (RLC) of myosin (Starr and Offer, 1978; Gruen and Gautel, 1999; Harris et al., 2011), Nag et al. (Nag et al., 2017) showed binding between MyBP-C and actin, MyBP-C and a proximal S2 fragment, S1 and S2, and S1 and either full-length MyBP-C or its N-terminal C0-C2 domains. Taken together, this experimental evidence supports the idea that human cardiac myosin exists in an equilibrium between open and sequestered states, where sequestered heads are functionally unavailable to interact with actin. The myosin motor domain can bind its proximal S2 tail leading to a sequestered conformation with decreased actin-activated ATPase activity, and this interaction can be regulated by post-translational modification such as phosphorylation (Trivedi et al., 2018; Nag et al., 2021). Furthermore, the MST data suggests that this equilibrium may also be governed by interactions between myosin and surrounding thick filament proteins.

In parallel with the development of structural evidence for a folded-back closed state of myosin, functional studies were also pointing to the existence of sequestered myosin heads in muscle. Using skinned skeletal muscle fibers, Cooke and his colleagues showed that there are three functional states of myosin in thick filaments, termed active, “disordered relaxed (DRX)” and “super relaxed (SRX)” states that have different ATP turnover lifetimes (Stewart et al., 2010; Cooke, 2011; Hooijman et al., 2011; McNamara et al., 2015). The active cycling state of myosin interacts with the thin filament and has a rapid ATP turnover of <1 s. The DRX state, in which myosin is detached from the actin and cycles ATP at a basal rate, turns over ATP in ∼30 s. Lastly, the SRX state has an ATP turnover time 10X slower than the normal basal rate, ∼300 s. This SRX state is found in both skeletal (Stewart et al., 2010) and cardiac (Hooijman et al., 2011) muscle; however, there is a key difference between the two. In skeletal muscle, upon activation by Ca^2+^ the SRX state is completely abolished, leading to maximal instantaneous force upon activation. In cardiac muscle, where there is a cyclical flux of Ca^2+^ with each heartbeat, the population of SRX myosin heads remains stable with Ca^2+^ activation. It was postulated that the SRX state in skeletal muscle serves to decrease the energy consumption of resting muscle while allowing rapid recruitment of myosin heads upon muscle activation, whereas the more stable cardiac myosin SRX population likely serves as a reserve pool of sequestered heads which conserves energy expenditure at rest but can be recruited when increased cardiac contractility is required (eg exercise).

In the original experiments done by the Cooke group, the relaxed skinned myofibers were initially incubated with a fluorescent ATP (mant-ATP), followed by a rapid chase with dark ATP (Stewart et al., 2010). The mant-ATP has increased fluorescence when bound to myosin, and a signal decay is observed as mant-ADP gets replaced by dark ATP. This is a single turnover experiment, the rate of which is limited by the phosphate release rate, which is slower than the mant-ADP release rate. The signal decay is fit to a double exponential, and the rate and proportion of the two different populations (DRX and SRX) are obtained. These populations had only been described in skinned striated muscle fibers where other sarcomeric proteins and other ATPase enzymes could potentially affect either the observed proportions or turnover rates. To determine whether these populations could be identified using purified myosin, the Spudich group developed a plate-based assay to study mant-ATP turnover using purified recombinant human cardiac protein (Anderson et al., 2018). Using the same HMM constructs with either 25-hep or 2-hep repeats of the proximal S2 tail, single nucleotide turnover assays of 2-hep HMM showed predominantly the DRX myosin population (∼80%) while 25-hep construct showed a majority of the population (60%) in the SRX state (Anderson et al., 2018). As this assay is done using purified myosin only, this observation is consistent with the notion that when the myosin cannot form a folded back (IHM-like) conformation (2-hep HMM), the majority of myosin is in the DRX state, while the myosin construct containing a long tail which presumably can form a folded back state will have a greater proportion of myosin heads in the SRX state. While this observation supports the correlation between the SRX functional state and the IHM structural state, it does not equate the two. Additional experimental perturbations that correlate the SRX and IHM (described below) strengthen the case that there is a causative association. However, it is worth emphasizing that in single turnover experiments the short S1 fragment of myosin, which has no proximal S2 to fold back onto, showed a small (10%) population of molecules in an SRX state, with a low ATP turnover rate (Anderson et al., 2018). As such, the SRX state of myosin cannot be exclusively defined as a folded-back state. Future (technically challenging) experiments combining single molecule FRET and simultaneous visualization of single molecule ATP turnover may provide information about both the structural and enzymatic state of myosin and thus lead to a better understanding of the relationship between SRX and IHM.

### Regulation of thick and thin filament activation

The myosin-actin interaction is the fundamental unit of force generation in the sarcomere, and other regulatory proteins modulate this process. MyBP-C is an important sarcomeric thick filament protein which binds to the myosin thick filament through MyBP-C’s C-terminal domains and to actin as well as the myosin head domain through MyBP-C’s N-terminal domains (Harris, 2021). MyBP-C has long been thought to work as either a “brake” for the sliding filament or an activator of the tropomyosin-troponin, reducing Ca^2+^ sensitivity of force and rates of force development (Flashman et al., 2004; Harris et al., 2011; Harris, 2021). Phosphorylation of MyBP-C by cAMP-dependent protein kinase (PKA) is thought to be a key element in regulating force production of the sarcomere (Kuster et al., 2012). As discussed earlier, MyBP-C has been known to interact with not just myosin, but also actin (Craig et al., 2014), myosin’s regulatory light chain (RLC) (Ratti et al., 2011), S1 without the RLC (Nag et al., 2017) and the proximal part of the S2 tail (Gruen and Gautel, 1999), and is an important determinant of sarcomere function. The thick filament is tied to the Z-disc by titin. Titin is the largest protein known in human cells, contains binding sites for many muscle-associated proteins, and is thought to sense the tension generated by the sarcomere (LeWinter and Granzier, 2013). Titin modulates the stiffness of the muscle, with its extensible I-band region acting as a molecular spring that develops passive force when the sarcomere is stretched during diastolic filling. As discussed later, myosin and MyBP-C comprise the vast majority of mutations responsible for HCM (Maron et al., 2012; Semsarian et al., 2015), while titin is now recognized as the gene most frequently mutated in patients with idiopathic DCM, but is rarely associated with HCM (Herman et al., 2012; LeWinter and Granzier, 2013; McNally et al., 2013).

On the thin filament side, the troponin complex, along with tropomyosin, has been identified to be the key regulator of actin-myosin crossbridge formation. In the heart, this regulatory unit converts increases in cytosolic Ca^2+^ concentration due to cardiac excitation into increased contractility of cardiac muscle by increasing the availability of the actin filament for myosin heads to bind and undergo a power stroke (so-called excitation-contraction coupling) (Kobayashi and Solaro, 2005). Troponin has three subunits, namely TnT, TnC and TnI. TnT interacts with tropomyosin, TnC binds to Ca^2+^ and TnI exerts an inhibitory effect on crossbridge formation by occupying the myosin-binding domain of actin (Parmacek and Solaro, 2004; de Tombe et al., 2010). There is one troponin complex to every seven actin monomers (Parmacek and Solaro, 2004). When Ca^2+^ binds to TnC, the whole troponin complex undergoes a conformational change, releasing TnI from the myosin-binding domain and allowing myosin heads access to the actin filament.

Ca^2+^ is the key regulator of thin filament activity, and its concentration is tightly regulated by Ca^2+^ release and reuptake in the sarcoplasmic reticulum (SR) by ryanodine receptor 2 (RyR2) and the sarco/endoplasmic reticulum-Ca^2+^-ATPase (SERCA2a), and by various voltage gated Ca^2+^ channels (Bers, 2002; Bers and Guo, 2005). Cytosolic Ca^2+^ concentrations oscillate between ∼10^–7^ M in diastole and up to 10^–5^ M during systole (Fearnley et al., 2011), and shift on the order of milliseconds (Bers, 2002), which allows appropriate cellular response to instantaneous changes in load. There is evidence for positive cooperativity in Ca^2+^ binding to TnC, which is part of the length-dependent thin filament activation seen in striated muscle (Bers, 2002; Bers and Guo, 2005; de Tombe et al., 2010). It has also been observed that the rate of force production continues to rise even after Ca^2+^ concentrations reach their peak. This may be ascribed to strong binding of myosin heads to the regulated thin filament, facilitating the movement of tropomyosin on nearby actin monomers away from the myosin binding site on actin, thus allowing the binding of heads in adjacent regions of the thin filament in a cooperative manner (Moss et al., 2004; Moss and Fitzsimons, 2010).

Thin filament activation can be controlled by phosphorylation of TnI, which decreases the affinity of TnC for Ca^2+^ and increases the off rate of Ca^2+^ from TnC (de Tombe et al., 2010). Phosphorylation of Ca^2+^ handling proteins, including phospholamban and RyR2, controls Ca^2+^ homeostasis in the myocyte, and can be disturbed in failing hearts which are under hyper-adrenergic stimulation (Wehrens and Marks, 2004). A recent report also suggests that the structure of the troponin complex in the thin filament is altered by increasing sarcomere length and is the main contributor to the increased Ca^2+^ sensitivity at low Ca^2+^ concentrations that is seen with length-dependent activation (Zhang et al., 2017). In contrast, the bulk of evidence suggests that conformational changes of myosin in the thick filament are responsible for increased force production in length-dependent activation (Zhang et al., 2017). There is also a report by Ait-Mou et al. (Ait-Mou et al., 2016) showing that titin strain mediates length-dependent activation *via* stretching of the sarcomere that causes structural rearrangements within both thick and thin filaments. Length-dependent activation of the sarcomere has been an active area of research for decades, and ongoing studies suggest that it involves important crosstalk between the thin and thick filaments (de Tombe et al., 2010)

Ca^2+^ sensitivity is an intrinsic property of the troponin complex, and it can be assessed, for example, by determining the pCa_50_ (−log [Ca^2+^] at half-maximal activation) of an enzymatic or motility assay over a range of Ca^2+^ concentrations (Kreutziger et al., 2007). A typical pCa curve involving regulated thin filaments is sigmoidal, with low activity on the left side of the curve at low Ca^2+^ concentrations (i.e. at high pCa value), followed by a steep increase in activity within a physiological Ca^2+^ concentration (pCa of 5–7), and plateauing of activity at high Ca^2+^ concentrations (i.e. at low pCa value). The pCa_50_ is the Ca^2+^ concentration that gives half-maximal activity. If the system is Ca^2+^ sensitized, the sigmoidal curve will shift to the left, and the pCa_50_ will increase (i.e. the system will show half-maximal activity at lower Ca^2+^ concentration). Functional assays incorporating these regulatory proteins that modulate the actin-myosin interaction are therefore extremely useful (Kobayashi and Solaro, 2005; Willott et al., 2010; Tardiff et al., 2011). The actin filament can be assembled with troponin-tropomyosin complexes to form a regulated thin filament *in vitro*, and can be used to perform all the *in vitro* assays used for actin-myosin analysis (Nag et al., 2015; Kawana et al., 2017). By using recombinant protein expression, functional human troponin and tropomyosin containing various disease-causing mutations can be expressed in bacteria (Sommese et al., 2013b; Gupte et al., 2015; Pan et al., 2015). Higher pCa_50_ values are seen in assays using regulated thin filaments comprised of tropomyosin or one of the troponin subunits containing an HCM-causing mutation, suggesting that the sarcomere is active at lower Ca^2+^ concentration and thus has higher Ca^2+^ sensitivity in these HCM patients (Sommese et al., 2013b; Gupte et al., 2015).

In addition to thin filament activation, there has been growing interest in the mechanism of activation of the thick filament through Ca^2+^ mediated processes (Ma et al., 2022). Ma et al. (Ma et al., 2022) used a small molecule inhibitor of the thin filament that enhances the Ca^2+^ off rate from troponin, and small-angle X-ray diffraction to show that Ca^2+^ progressively moves the myosin heads from an ordered “off” state on the thick filament backbone to a disordered “on” state closer to the thin filament. When reconstituted cardiac synthetic thick filaments were used to assess the SRX population with either a single turnover assay or a basal ATPase activity assay under varying Ca^2+^ concentrations, there was a Ca^2+^-dependent decrease in SRX proportion and increase in basal activity (Ma et al., 2022). This Ca^2+^-dependent activation was not seen in purified HMM with varying tail lengths (all lacked the filament-forming light meromyosin: LMM), and it is speculated that the presence of LMM and assembly into bipolar thick filaments is necessary for this property. The authors also tested adding saturating amounts of free Mg^2+^, which would bind to the Ca^2+^/Mg^2+^ binding site of RLC, and saw no change in activation suggesting that the RLC motif is not the primary Ca^2+^ transducer responsible for the off-to on-transition in the thick filament (Ma et al., 2022).

Myocytes containing mutated sarcomeric proteins or treated with small molecules that shift the pCa_50_ curve to the left contract at lower Ca^2+^ concentrations and do not fully relax at physiological diastolic Ca^2+^ levels (Fraysse et al., 2012; Marston, 2016). While it is not clear whether the changes seen at the protein level using *in vitro* assays always translate into similar findings at the cell or tissue level, activation of the sarcomere at lower Ca^2+^ levels may have important implications for the pathophysiology of hypertrophic cardiomyopathy, which is characterized by the development of diastolic dysfunction in the early stages of the disease. Ho et al. reported that patients who were carriers of HCM mutations in sarcomere genes, but who were yet to develop significant hypertrophy, still had echocardiographic evidence of diastolic dysfunction as well as increased systolic function (Ho et al., 2002). The diastolic dysfunction in this cohort cannot be attributed to either hypertrophy or to the myocardial fibrosis that typically accompanies it, suggesting that the primary factor driving this early diastolic dysfunction is likely found at the sarcomere level where actin-myosin crossbridges are being formed.

## Genetic alteration of F_ensemble_ parameters leads to HCM

We now consider the effect of mutations in sarcomeric proteins that cause cardiovascular disease. Since the discovery of the R403Q mutation in MYH7 as the first HCM-causing mutation by the Seidman group (Geisterfer-Lowrance et al., 1990), it has been well established that HCM is largely a disease of the sarcomere, except for a few phenocopies such as glycogen storage disease and amyloidosis (Seidman et al., 2011). Linkage analysis and candidate gene screening in families and sporadic cases have led to the discovery of disease-causing mutations in genes encoding many other sarcomeric proteins, including MyBP-C, the troponin complex and tropomyosin (Marston, 2011; Moore et al., 2012; Fatkin et al., 2014). Functional characterization of the effects of these HCM mutations has been a long-time challenge in the field, especially for myosin mutations: for example, earlier studies of cardiac myosin purified from human cardiac biopsy samples have shown conflicting results in *in vitro* motility assays (CUDA et al., 1997; Palmiter et al., 2000). Biopsy material suffers from limited availability, typically comes from patients with late stage disease, is a mix of wildtype and mutant protein as virtually all patients are heterozygous for their disease-causing mutations (Helms et al., 2014), and requires careful handling to preserve enzymatic function. Therefore, a number of animal models harboring HCM mutations in their corresponding genes have been generated and studied.

The best-characterized model is the transgenic mouse carrying the R403Q mutation in α-cardiac myosin (Geisterfer-Lowrance et al., 1996), which is the predominant ventricular isoform in small rodents. At age 30-week the mouse recapitulated human HCM pathophysiology and histopathology (Geisterfer-Lowrance et al., 1996; Georgakopoulos et al., 1999). Mutant α-cardiac myosin purified from ventricular tissue showed increased ATPase activity and actin filament gliding velocity by *in vitro* motility assays (Tyska et al., 2000), and increased ensemble force, but no change in intrinsic force (Tyska et al., 2000; Debold et al., 2007). However, seminal work by Susan Lowey and others showed that when the same mutation was introduced into the mouse β-cardiac myosin backbone, there was no significant change seen in actin velocity, and there was a slight decrease in ATPase activity (Lowey et al., 2008). In keeping with the *in vitro* motility results, the ADP release rate was 20% higher for α-cardiac myosin with the R403Q mutation while there was no change with the same mutation in the β-cardiac myosin background (Fatkin et al., 2014). There are over 80 amino acid residue differences between mouse α-cardiac myosin and human β-cardiac myosin (Weiss et al., 1999), and there are considerable functional differences between α- and β-cardiac myosin in ATPase activity and motility (Deacon et al., 2012; Aksel et al., 2015). In other words, while the R403Q mutation in α-cardiac myosin likely shows hypertrophy in mouse models due to increased ATPase activity and ensemble force leading to hypercontractility, the R403Q mutation in human β-cardiac myosin may cause hypertrophy through entirely different changes to myosin biomechanics. Taken together, these observations highlight the difficulty in using mouse models to study disease-causing mutations in MYH7 and the importance of using the appropriate myosin backbone to accurately determine the effects of these mutations on myosin function.

### Functional assessment of HCM mutations using purified recombinant human cardiac myosin *in vitro*

For many years there has been a gap in knowledge between the identification of disease-causing mutations and how these mutations lead to the secondary cellular events that cause hypertrophic signaling (Sivaramakrishnan et al., 2009). Model systems such as transgenic mice that recapitulate HCM morphology have been important in understanding the cell biology of hypertrophy, while the exact mechanism of the index signal that triggers the hypertrophic signaling has been lacking. *In vitro* expression of enzymatically active recombinant cardiac myosin had been challenging until an expression system was developed using the C2C12 mouse myoblast cell line (Srikakulam and Winkelmann, 2004; Liu et al., 2008; Resnicow et al., 2010). Using this approach, we and others are now able to obtain highly purified, functional recombinant human β-cardiac myosin with engineered disease-causing mutations.

The overarching hypothesis here is that HCM mutations result in increased power output (Anan et al., 1994) by altering the contractility parameters discussed in part 1 above. As mentioned earlier, power is the product of force and velocity, and is also represented by the area under the curve of the force vs. velocity relationship of muscle contraction. This hypothesis is based on the clinical observation that HCM patients often present with hyperdynamic physiology (Klein et al., 1965; Wilson et al., 1967; Seidman et al., 2019), and the earliest signs of HCM pathology are abnormal diastolic function and supranormal ejection fractions suggestive of a hypercontractile left ventricle (Ho et al., 2002).

The first reported biomechanical analyses of recombinant human β-cardiac myosin containing HCM-causing mutations led to confusing and inconclusive results. The first experiments focused on **R453C** (Sommese et al., 2013a), which is one of the earliest identified mutations known to cause a severe clinical phenotype (Watkins et al., 1992). Similar to prior mouse work (Palmer et al., 2004; Debold et al., 2007), the R453C mutation in human β-cardiac myosin caused a 50% increase in F_intrinsic_, ∼30% decrease in both ATPase activity and actin gliding velocity, and thus no significant change in duty ratio (Sommese et al., 2013a). By simple calculations, the overall ensemble force is expected to be increased, and the power output also increased despite a 30% decrease in velocity. This finding was consistent with the hypothesis that an increase in power output leads to HCM. However, in contrast to the steady-state experiments, transient kinetic studies of the R453C mutant protein by Michael Geeves’ group revealed that surprisingly few parameters were altered by the mutation, with the exceptions being a 35% reduction in ATP binding to the motor domain and a three-fold slowing of the ATP hydrolysis step/recovery stroke, which could become the rate-limiting step for the ATPase cycle in place of the phosphate release step (Bloemink et al., 2014).

Next, the **R403Q** mutation was rigorously characterized (Nag et al., 2015). When assayed using actin filaments, the R403Q myosin resulted in an ∼15% reduction in F_intrinsic_, two-fold reduction in actin-binding affinity, and ∼10% increase in unloaded actin gliding velocity compared to wildtype (WT) (Nag et al., 2015). There was no significant change in ATPase activity (= 1/t_c_), step size (d), or ADP release rate (which affects t_s_). The loaded *in vitro* motility assay showed characteristics of lower contractility at higher external loads. With RTFs, however, there was no increase in the unloaded velocity of gliding filaments, and an ∼30% lower ATPase activity (Nag et al., 2015). In addition to the interesting differences between actin-based vs. RTF-based assays, it was quite surprising to see an overall decrease of contractile parameters with the R403Q mutation.

Further investigations were performed on well-known HCM mutations in the converter region of myosin, which has been known as a hot-spot for HCM mutagenesis (Colegrave and Peckham, 2014; García-Giustiniani et al., 2015; Homburger et al., 2016). We chose three mutations that were known to be severely pathogenic, namely **R719W** (Anan et al., 1994), **R723G** (Enjuto et al., 2000) and **G741R** (Fananapazir et al., 1993). Given that converter movement is coupled to major mechanical changes including the power stroke, as well as biochemical events including load-dependent ADP release, ATP hydrolysis and phosphate release, we expected major alteration in these parameters for these HCM mutations. In contrast to our prediction, we did not see significant increases in either enzymatic or biophysical properties: R719W and R723G resulted in 15–30% reductions in F_intrinsic_ and ∼15% increases in velocity, while ATPase activity was unchanged. Loaded *in vitro* motility showed characteristics of a trend toward lower contractility. Meanwhile, G741R did not show significant changes compared to WT in any of the parameters tested. Unlike the R403Q mutation, which sits near the actin-binding domain, there was no difference in the results between actin-based or RTF-based assays.

In summary, the biochemical and biophysical effects of these HCM mutations at the molecular level did not support the clinically observed hypercontractility, leading us to suspect an alternative mechanism as discussed below.

### Myosin availability as a primary mechanism for hypercontractility in HCM

As mentioned earlier, a relatively flat surface of the myosin motor domain termed the “myosin mesa” was noted for its high proportion of highly conserved residues across different species. In addition, this surface turned out to be enriched in HCM mutations (Spudich, 2015; Homburger et al., 2016). The myosin mesa has a cluster of positively charged (predominantly arginine) residues, most of which, when mutated, cause HCM. Homburger and others searched a large HCM patient registry database and mapped the location of mutations in cardiac myosin (Homburger et al., 2016) and found that the myosin mesa, converter domain and proximal S2 tail domain are three hot spots for HCM mutations in the myosin molecule. The converter domain and proximal S2 tail domain have been previously implicated in the stability of the IHM folded back state (Wendt et al., 1999; Blankenfeldt et al., 2006) and the positively-charged myosin mesa is an attractive site for binding other protein surfaces, either intramolecularly or intermolecularly (for example with MyBP-C) (Trivedi et al., 2018). Therefore, it was hypothesized that any mutations in these areas might be expected to weaken either inter- or intra-molecular interactions important for forming the folded back state, thereby pushing the equilibrium to more myosin heads in an “on state” and increasing the number of myosin heads functionally accessible (N_a_) for interaction with actin (Trivedi et al., 2018; Spudich, 2019; Nag et al., 2021).

Nag et al. (Nag et al., 2017) first tested this hypothesis by studying three mutations that lie on the myosin mesa surface (**R249Q, H251N** and **R453C**) and one in proximal S2 (**D906G**), which were hypothesized to disrupt head-tail interactions as described above. Strikingly, all four mutants significantly weakened the affinity of proximal S2 for short S1^15,48^. In contrast, three HCM mutations lying outside of these areas (R403Q, D239N and R870H) did not have any effect on proximal S2 binding affinity, as studied using MST (Adhikari et al., 2016; Nag et al., 2017). The four mutations that showed weakened affinity for S2 are predicted to open folded-back heads to become functionally available, hence increasing N_a_.

As mentioned above, HMM constructs were developed with both long and short proximal tail domains (Nag et al., 2017) which show differing abilities to form the sequestered state. Utilizing these 2-hep and 25-hep HMM constructs, Adhikari et al. (Adhikari et al., 2019) studied the R249Q and H251N mutations, which are thought to affect residues involved in the S1-S2 interface of the folded-back state, along with the converter domain mutation R719W and a mutation (D382Y), both of which are located at the S1-S1 interface in homology models of folded back β-cardiac myosin. The mant ATP single nucleotide turnover experiment using H251N or R249Q mutant 25-hep HMM resulted in significant decreases (41% and 59%, respectively) in the number of SRX heads as compared to WT. The increase in the DRX/SRX ratio also corresponded to an increase in actin-activated ATPase activity of the mutant 25-hep HMMs, suggesting that the mutations cause more heads to be functionally accessible to actin and thus increased the ensemble enzymatic activity. R719W and D382Y also increased the percentage of DRX heads in the context of 25-HMM constructs, and increased actin-activated ATPase activity (k_cat_) as well. In particular, the k_cat_ of R719W 25-hep HMM was essentially the same as R719W 2-hep HMM, suggesting that the R719W mutation had a strong effect on opening up the heads of folded-back 25-hep HMM molecules and made these heads accessible for interaction with actin. This significant increase in N_a_ would likely dominate the minor decreases in F_intrinsic_ and duty ratio parameters measured previously to drive an increase in ensemble force (Kawana et al., 2017). Indeed, all four HCM-causing mutations affecting residues located at putative interfaces in the folded back state appeared to cause an increase in N_a_ as the primary driver of hypercontractility.

Interestingly, Adhikari et al. (Adhikari et al., 2019) also tested an HCM-causing mutation, I457T, that affects a residue in the transducer region of the motor that is remote from putative head-head or head-tail interfaces. They found that the mutated 25-hep HMM had an SRX/DRX ratio indistinguishable from WT 25-hep HMM in single turnover experiments and a similar ratio of 2-hep:25-hep actin-activated ATPase activity as WT, suggesting that this mutation did not affect N_a_. Instead, I457T caused a ∼75% increase in the k_cat_ and a greater than 2-fold increase in the *in vitro* sliding velocity compared to WT controls. This mutation illustrates that increases in myosin motor function alone can drive hypercontractility without affecting N_a_. However, I457T is unique in not increasing N_a_ among the >20 HCM-causing mutations we have studied in the context of the 2-hep and 25-hep HMM constructs, and may well be the exception that proves the rule.

Sarkar et al. (Sarkar et al., 2020) studied the **R403Q** and **R663H** mutations using the 2-hep and 25-hep HMM constructs. The R663H mutation is a well-known HCM-causing mutation and the initial clinical report of it showed a strong correlation between the mutation and the development of atrial fibrillation (Gruver et al., 1999). Interestingly, a short S1 construct with the R663H mutation showed no difference compared to WT in terms of ATPase, *in vitro* motility, or single molecule force measurements. There was no difference in the binding affinity of short S1 and proximal S2 between WT and R663H or R403Q using MST, which might be expected given that neither R663 nor R403 are predicted to be in direct contact with proximal S2 in the folded back state. However, when R663H 25-hep and 2-hep HMM were analyzed with single nucleotide turnover and actin-activated ATPase assays, R403Q 25-hep HMM and R663H 25-hep HMM showed significantly higher DRX percentages and higher ATPase activity than WT 25-hep HMM, suggesting that these mutations destabilize the folded back state and provide more myosin heads for interaction with actin. Sarkar et al. (Sarkar et al., 2020) also evaluated the effect of adding the large N-terminal fragment of MyBP-C (C0C7 domain) and showed that binding of the purified C0C7 fragment to WT 25-hep HMM led to an increase in the percentage of myosin heads in the SRX. The R403Q mutation, however, abolished the binding of 25-hep HMM to the C0C7 fragment, and the ratio of SRX/DRX in single turnover assay was similar with or without the addition of C0C7. In contrast, the R663H 25-hep HMM did bind C0C7 with an affinity similar to that of WT; despite that, the SRX/DRX ratio of R663H 25-hep HMM was unaltered by the presence of C0C7. Taken together, these observations suggest that while MyBP-C can bind to heads that are not in the auto-inhibited SRX state, such binding does not necessarily cause those heads to adopt the SRX state. Therefore, some MYH7 mutations like R663H may increase N_a_ despite binding to MyBPC, thus escaping this mechanism of thick filament regulation (Sarkar et al., 2020).

Finally, Morck et al. (Morck et al., 2022) reported the effect of five HCM-causing mutations in the myosin lever arm. These included mutations in the pliant region (D778V, L781P, and S782N), the bent region between the light chains (A797T), and the hook joint (F834L). Using the actin-activated ATPase assay, they found that 2-hep HMM with any of these five mutations had a similar k_cat_ to WT 2-hep, while 25-hep HMM containing any of these mutations showed a relative increase in k_cat_ compared to WT 25-hep, demonstrating that these mutations caused more heads to be available to interact with actin. Interestingly, the three pliant region mutations did not lead to a decrease in the SRX population in the single turnover assay, emphasizing that the IHM state and the SRX state cannot always be equated. In this case, it’s possible that the autoinhibition of the myosin is disrupted only in the presence of actin. These three mutations also caused a variable impact on duty ratio, ensemble force and power output at the single molecule level. On the other hand, the light chain binding region mutations (A797T and F834L) led to both an increase in k_cat_ of the actin-activated myosin ATPase compared to 25-hep WT HMM and a significantly reduced SRX population in the single turnover assay (and had no effects on basic biomechanical parameters). Specific light chain positioning is likely required to access the folded state, and these mutations may act primarily by disrupting that positioning. The lever arm mutations highlight the importance of assessing all aspects of myosin function using multimodal assay systems.

### Early vs. late onset HCM mutations

All of the above MYH7 mutations are observed predominantly in patients with adult-onset HCM. Adhikari et al. (Adhikari et al., 2016) studied mutations that are found predominantly in the pediatric population and thus termed “early-onset” HCM (Kaski et al., 2009). Two mutations were chosen - **H251N** on the “myosin mesa” and **D239N** within the Switch-1 (nucleotide-binding) domain, and both were studied in the context of short S1. Both mutations significantly increased ATPase activity, 24% for H251N and 50% for D239N compared to WT (Adhikari et al., 2016). F_intrinsic_ was increased 46% for H251N and 23% for D239N, and significant increases in actin gliding velocity were seen as well (94% for D239N and 40% for H251N) (Adhikari et al., 2016). Loaded *in vitro* motility assay experiments showed an upward shift in the load-velocity curve suggestive of increased ensemble force compared to WT (Adhikari et al., 2016). These striking changes contrast significantly compared to five mutations seen predominantly in adult patients (R403Q, R663H, R719W, R723G, G741R) and may be the basis for the early-onset, more severe phenotype compared to the more typical adult-onset disease.

However, when Vera et al. (Vera et al., 2019) compared short S1 constructs containing either early (H251N, D382Y, P710R and V763M) or adult onset (R719W, R723G and G741R) HCM mutations by measuring steady-state and transient kinetics, there was no clear difference in the degree of changes in these parameters. There was also no unifying direction of changes in any of the kinetic parameters. The H251N mutation had significant increases in the above parameters, while the other mutations showed only modest changes (Vera et al., 2019). Interestingly, one of the HCM mutations, P710R, shared properties with the DCM-causing mutations (Ujfalusi et al., 2018), including reduced ATPase activity (k_cat_), lower occupancy of the force holding actin-myosin-ADP state, a lower duty ratio and a more economical use of ATP for both rapid movement and force generation (Ujfalusi et al., 2018).

Given the seemingly inconsistent findings for the kinetics of the P710R myosin, this mutation was studied further (Vander Roest et al., 2021). Using optical trapping with harmonic force spectroscopy (HFS), the single molecule properties of P710R short S1 showed reduced load sensitivity and a decrease in the step size. The velocity of actin filament gliding was also reduced. Based on actin-activated ATPase rates and load-dependent actin detachment rates, it was calculated that P710R reduced the duty ratio especially at higher loads. Similar to the results from the kinetic studies, these findings using the short S1 construct suggested that the P710R mutation should result in hypocontractility. However, when P710R 2-hep HMM and 25-hep HMM were studied using the actin-activated ATPase assay, the P710R mutation did not result in the ∼40% decrease in k_cat_ between 2-hep and 25-hep HMM which is seen with the WT constructs. The single turnover assay showed that P710R significantly reduced the proportion of myosin in the SRX state down to 27%, close to the SRX proportion for the 2-hep HMM, which is around 20%. These findings suggest that the P710R mutation results in a significant disruption of the SRX state, leading to more myosin heads functionally available. As for R403Q and R719W, this significant increase in N_a_ may compensate for the hypocontractile features seen in other assays, resulting in a net increase in ensemble force leading to hypercontractility.

### Multiscale effects of HCM mutations

In order to understand which effects (hypercontractile vs. hypocontractile) of the P710R mutation on myosin function predominate in an ensemble, an induced pluripotent stem cell-derived cardiomyocyte (iPSC-CM) line carrying the P710R mutation in one allele of MYH7 was generated using CRISPR/Cas9 gene editing and the resultant cells were micropatterned on substrates of appropriate physiological stiffness to obtain cardiomyocytes containing well-aligned myofibrils (Ribeiro et al., 2015; Vander Roest et al., 2021). Traction force microscopy showed that the iPSC-CM with P710R β-cardiac myosin had significantly higher contractile force, with increased peak force and contraction time. The transmission electron microscopy image of also showed significantly disrupted myofibril organization when compared to isogenic controls. The cell size was also increased, and ERK and Akt pathways were activated more than in control cells. Further computational modeling integrating the biochemical and biophysical parameters predicted the measured increase in traction forces, highlighting the effect of myosin availability (N_a_) as a major molecular determinant of hypercontractility in HCM.

The effect of other HCM mutations in myosin on N_a_ have also been studied using iPSC-CMs. Toepfer et al. (Toepfer et al., 2019) reported the effect of pathogenic MYH7 variants (R403Q, V606M and R719W), which resulted in decreased SRX fractions and enhanced cardiomyocyte contractility. They also showed that HCM-causing mutations in MYBPC3 (the gene encoding MyBP-C), as well as stepwise loss of MYBPC, resulted in decreases in the SRX population and increases in cardiomyocyte contractility. The effect of MyBP-C loss was attenuated by introducing a DCM variant (F764L) of MYH7 or a myosin inhibitor (MYK-461) (Toepfer et al., 2019). The effect of loss of MyBP-C on myosin function observed in the cellular model highlights the key role MyBP-C plays in the modulation of myosin function and overall sarcomere activity. It is also a proof of principle that a myosin inhibitor is capable of attenuating the overall cellular contractility in HCM due to non-MYH7 variants (Toepfer et al., 2019).

## Myosin modulation as a therapeutic tool for treating cardiomyopathy

### Myosin inhibitors for HCM

Based on the hypothesis that HCM is fundamentally due to hypercontractile function at the sarcomere level, a small molecule to inhibit myosin activity was developed as a proof of principle to treat HCM. Mavacamten (MYK-461) was developed (Green et al., 2016) and tested in three different HCM mouse models (R403Q (Geisterfer-Lowrance et al., 1996), R453C (Palmer et al., 2004) and R719W (Teekakirikul et al., 2010)). Early treatment of pre-hypertrophic mice with MYK-461 prevented development of hypertrophy, and treatment of older mice with existing hypertrophy reversed the increased wall thickness (Green et al., 2016). The molecular mechanism of mavacamten was reported by Anderson et al. (Anderson et al., 2018). They used negative stain EM to show that mavacamten stabilized a folded back state of 25-hep human β-cardiac HMM, and used the single nucleotide turnover assays to show in parallel that it also increased the fraction of heads in the SRX state (Anderson et al., 2018). The ability of mavacamten treatment to increase the SRX population was also seen in skinned cardiac fibers from an R403Q heterozygous pig model and a biopsy sample from a patient carrying the R663H mutation. At baseline, the skinned cardiac fiber from the R403Q pig had reduced SRX population (16% vs. 26% in WT pig), and mavacamten treatment restored the SRX population up to 30%. In addition, the maximum tension in the skinned pig fiber was reduced after treatment with mavacamten (Anderson et al., 2018). Mavacamten also caused an increase in the ordering of myosin heads along the backbone of the thick filament, as observed by the substantial increase in the myosin-based helical layer line reflections in low-angle X-ray diffraction images (Anderson et al., 2018). Taken together, mavacamten reduced the population of functionally available myosin motors (N_a_) in the sarcomere by stabilizing a folded back state of myosin, and the resultant reduction in F_ensemble_ led to prevention and/or reversal of hypertrophy in HCM mouse models. Mavacamten was subsequently tested in a series of clinical trials with HCM patients.

The phase I study was completed and showed good safety and tolerability in healthy volunteers and a small number of patients (Maron et al., 2016). The phase II study was conducted in patients with obstructive HCM who had significant LVOT gradients due to the hypertrophied septum with resultant heart failure symptoms such as exertional dyspnea (Heitner et al., 2019) (*see* Introduction and Figure 1). The mavacamten treatment resulted in a dramatic decrease in LVOT gradient down to the normal range, and after the treatment period concluded, the LVOT gradients returned to pre-treatment baselines (Heitner et al., 2019). In the phase III study of obstructive HCM patient (EXPLORER-HCM), mavacamten showed improvement in the composite endpoint of exercise capacity and symptom severity (using the New York Heart Association (NYHA) functional classification) (Olivotto et al., 2020). This is the first medication for HCM that showed a benefit in a randomized controlled trial. Mavacamten was also studied in a phase II study for non-obstructive HCM patients (MAVERICK-HCM). This trial showed no significant change in the above composite endpoint, while the biomarker for ventricular stretch was significantly reduced (Ho et al., 2020). It is worth noting that the participants in these trials did not necessarily carry MYH7 variants, and thus the effect of mavacamten is exerted by bringing down the net F_ensemble_ which leads to normalization of sarcomere function.

Currently a long-term extension study for EXPLORER-HCM and MAVERICK-HCM participants is under way (MAVA-LTE), to study the long-term effects of this medication (Rader et al., 2021). For the former EXPLORER-HCM participants, mavacamten showed durable improvement in reduction of LVOT gradients, diastolic function, NT-proBNP (biomarker for ventricular stretch) and NYHA functional class (assessment of subjective symptoms) (Rader et al., 2021). The imaging study of the EXPLORER-HCM participants also showed significant changes in HCM morphology. The MRI study showed reductions in cardiac mass and wall thickness (Saberi et al., 2021), and the echocardiographic analysis also showed improvement in left ventricular diastolic function parameters and left atrial size (Hegde et al., 2021). The participants in these trials have already developed hypertrophy, and thus these imaging studies suggest that the effect of myosin inhibition to reduce hypercontractility at the sarcomere level has a downstream effect on hypertrophic processes resulting in at least partial remodeling of existing hypertrophied myocardium. Based on the result of EXPLORER-HCM, mavacamten was recently approved by the FDA for the treatment of patients with obstructive HCM.

A newer generation myosin inhibitor was developed by Cytokinetics and has been studied in obstructive HCM patients (Chuang et al., 2021). Aficamten has a shorter half-life and less drug interactions than mavacamten. A phase II study was reported last year, and showed significant reductions in LVOT gradient with a return to the pre-treatment baseline when the therapy was stopped (Maron, 2021). Currently, a phase III study is being conducted, mainly focusing on exercise capacity assessed by cardiopulmonary exercise testing.

### Myosin activators for systolic heart failure

Although this review is primarily focused on HCM molecular pathophysiology and the pharmacotherapy for HCM that has been developed under the hypothesis that HCM is fundamentally a hyperactive sarcomeric disease, the opposite end of the spectrum - systolic heart failure where the contractile function of the heart is compromised due to various underlying etiologies, is worthy of mention. Malik and others performed a small molecule screen using thin-filament activated ATPase activity of β-cardiac myosin, and developed **omecamtiv mecarbil (OM)** as the first cardiac myosin activator (Malik et al., 2011). OM showed increases in cell length shortening without changing Ca^2+^ transients in isolated rat cardiac myocytes. OM also improved echocardiographic and hemodynamic parameters using a dog heart failure model, primarily through increases in systolic ejection time, without changes in the rate of LV pressure development (dP/dt). Interestingly, OM seems to increase RTF-activated cardiac myosin ATPase activity at lower Ca^2+^ concentrations up to pCa 6, at which point a crossover occurs and at higher concentration it actually reduces the ATPase activity (Malik et al., 2011). Subsequent reports of RTF-activated porcine cardiac myosin ATPase activity (Marston, 2011) and actin-activated human cardiac myosin ATPase activity (Fatkin et al., 2014) both showed decreased ATPase activity at OM concentration of 10 and 100 μM, respectively. From this work, the binding site for OM was speculated to be a cleft in a region where the relay helix and the converter domain converge at the base of the lever arm (Malik et al., 2011).

OM was studied in a series of clinical trials (Cleland et al., 2011; Teerlink et al., 2011; Teerlink et al., 2016a; Teerlink et al., 2016b), and showed increases in systolic ejection time, ejection fraction and stroke volume on echocardiographic assessment (Cleland et al., 2011; Teerlink et al., 2011; Teerlink et al., 2016a). OM recently completed a phase III randomized, double-blind, placebo-controlled clinical trial in 8,000 systolic heart failure patients (Teerlink et al., 2021). The primary endpoint of the study was a composite of cardiovascular death and heart failure hospitalization, and OM met the primary endpoint, though the effect was small (8% relative risk reduction). Interestingly, subgroup analysis showed that the sicker patients (more advanced symptoms or lower LVEF) had more benefits from OM therapy.

There have been many investigations on the exact mechanism of sarcomere activation by OM. Using recombinant human cardiac myosin motor domain fused to GFP, Winkelmann et al. (Winkelmann et al., 2015) reported that OM binds in a narrow cleft that separates the N-terminal 25-K domain from the lower portion of the 50-K domain of the motor domain, which is involved in coupling structural elements that are linked to the rotation of the lever arm into the PPS conformation. Rohde et al. (Rohde et al., 2017) investigated the effect of OM on the kinetics of the myosin powerstroke using a combination of transient time-resolved FRET and transient biochemical assays. By measuring the FRET signal between a fluorescent donor on the RLC domain and fluorescent nucleotide, the group dissected the steps of actin binding, the powerstroke, and phosphate release. They concluded that in the absence of OM, myosin binds to actin, undergoes the powerstroke, and then releases phosphate, which is the rate-limiting step of the ATPase cycle. However, whether phosphate release occurs before or after the stroke is still a matter of debate (Houdusse and Sweeney, 2016; Planelles-Herrero et al., 2017) In the presence of OM, the phosphate release rate is increased; however the overall ATP turnover rate was slowed, owing to even greater slowing of the actin-induced rotation of the myosin light chain binding domain (Rohde et al., 2017). There was no change in ADP release rate from a post-stroke state (Liu et al., 2015), and the rate-limiting step for the steady-state ATPase cycle now became the actin-induced rotation of myosin in the presence of OM. This results in the accumulation of a prolonged actin-bound state of the myosin which might act as a load in the contracting sarcomere (Rohde et al., 2017), but might also serve to induce cooperative binding of more myosin heads to actin by its effect on the tropomyosin-troponin system, thus activating the sarcomere.

A similar conclusion was reached by Swenson et al. (Swenson et al., 2017) using recombinant human cardiac myosin and transient kinetic assays that showed a slow product release pathway, resulting in the accumulation of non-force generating heads (Swenson et al., 2017). The accumulation of a state of myosin that undergoes prolonged binding to actin is likely the reason for significant slowing of gliding actin filament velocities in *in vitro* motility assays in previous reports (Aksel et al., 2015; Liu et al., 2015; Winkelmann et al., 2015; Swenson et al., 2017). Liu et al. (Liu et al., 2018) used HFS technology to study the load-dependent detachment of myosin from the actin filament. In the presence of OM, myosin’s detachment rate at zero load (k_0_), the distance to the transition state (a measure of force sensitivity, δ), and the myosin stroke size were all reduced. Woody et al. (Woody et al., 2018) used a feedback-controlled laser trap and observed a similar effect of OM causing the detachment rate to become independent of both applied load and ATP concentration (Woody et al., 2018). The decrease in detachment rate was manifest as slowing of actin gliding velocity using *in vitro* motility assays (Aksel et al., 2015; Liu et al., 2018) and as reduced cardiomyocyte contractility (Kampourakis et al., 2018). Taken together, these findings suggest that OM binding to myosin results in a myosin head that attaches to the actin filament but does not produce a full functional stroke (Spudich, 2019).

The effect of OM at the cellular and organ level was rather surprising, given that OM appears to inhibit the individual myosin from stroking and hence should reduce contractility (Liu et al., 2018). The activation effect of OM is seen at the whole sarcomere level when only a fraction of the myosin heads are bound to OM. As discussed above, activation of the myocyte is likely due to activation of the thin filament by the OM-bound myosin, which has a prolonged binding to the thin filament, promoting more non-OM-bound myosin heads to interact with actin, leading to myocyte activation (Malik et al., 2011). This is a distinct way of increasing the ensemble force. This type of activation has been seen in HCM mutations affecting thin filament components, including the troponin complex (Sommese et al., 2013b), which resulted in an increase in Ca^2+^ sensitivity and activation of the thin filament at lower [Ca^2+^]. It is notable that OM had a similar effect on thin filament activation *via* interaction with myosin and not the thin filament components. Overall, the mechanism of OM highlights the complexity of myosin function in the sarcomere and the potential for pharmacological modulation.

## Future perspectives and conclusion

The fundamental mechanisms underlying HCM pathogenesis have long been studied using a variety of approaches, but it is only in the last 10–15 years that the field has advanced to using engineerable human proteins and cells to accurately study the effects of HCM-causing mutations. Using expressed and purified human β-cardiac myosin containing the ventricular human cardiac light chains, extensive work has led to the conclusion that a majority of HCM mutations cause hypercontractility of the heart by shifting myosin molecules from an off-state to an on-state, which results in an increase in N_a_, the number of heads functionally available for interacting with actin (Table 1).

**TABLE 1:** Summary of the functional effect of HCM mutations in cardiac myosin.

HCM mutation	Intrinsic force (F_intrinsic_)	Velocity (v)	ATPase (k_cat_)	Number of available myosin heads (N_a_)	References
Change from wildtype human β-cardiac myosin					
R403Q	↓	↑	↑	↑	Nag et al. (2015), Nag et al. (2017), Sarkar et al. (2020)
R453C	↑	↓	↓	-	Sommese et al. (2013), Nag et al. (2017), Bloemink et al. (2014)
R719W	↓	↑	NC	↑	Kawana et al. (2017), Adhikari et al. (2019)
R723G	↓	↑	NC	-	Kawana et al. (2017)
G741R	NC	NC	NC	-	Kawana et al. (2017)
R663H	NC	NC	NC	↑	Sarkar et al. (2020)
R249Q	-	↓	↓	↑	Nag et al. (2017), Adhikari et al. (2019)
I457T	-	↑	↑	NC	Adhikari et al. (2019)
P710R	-*	↓	↓	↑	Vander Roest et al. (2021), Vera et al. (2019)
V763M	-	↑	NC	-	Vera et al. (2019)
H251N	↑	↑	↑	↑	Adhikari et al. (2016), Nag et al. (2017), Adhikari et al. (2019), Vera et al. (2019)
D239N	↑	↑	↑	↑	Adhikari et al. (2016)
D778V	↓**	↑	↑	↑	Morck et al. (2022)
L781P	NC**	↓	NC	↑	Morck et al. (2022)
S782N	↓**	NC	NC	↑	Morck et al. (2022)
A797T	-	NC	NC	↑	Morck et al. (2022)
F834L	-	NC	NC	↑	Morck et al. (2022)

Summary of HCM mutations that have been studied using purified human β-cardiac myosin heavy chain containing human ventricular cardiac light chains. While F_intrinsic_, v and k_cat_ values showed no consistent trends among the HCM variants, the N_a_ was increased in all tested variants except one. NC, no change; dash, not determined. * The intrinsic force has not been measured, while optical trapping using harmonic force spectroscopy assay showed reduced step size of the myosin motor and reduced load sensitivity of the actin detachment rate at the single molecule level. ** The intrinsic force has not been measured for this mutation. Optical trapping using harmonic force spectroscopy was used to obtain load-dependent detachment rate, load sensitivity and step size. The average force of the sarcomere was calculated using these parameters.

In 2018, Robert-Paganin, Auguin and Houdusse (Robert-Paganin et al., 2018) reported important structural data using an optimized quasi-atomic model of the folded back IHM state of bovine cardiac myosin, coupled to in silico analysis of the effects of 178 HCM mutations previously described (Robert-Paganin et al., 2018). They suggested that the formation of the IHM requires that both heads adopt an asymmetric conformation while the two motor domains position the lever arm up as in the PPS state. According to their prediction, a majority of the mutations, about two-thirds, would lead to destabilization of the IHM, increasing N_a_. Out of these mutations, roughly half were not located at the interfaces of the IHM (head-head, or head-tail), but are predicted to alter the stability of the PPS conformation that is necessary to form the IHM (Robert-Paganin et al., 2018). The fraction of HCM mutations that are primarily increasing N_a_ could be higher than this. The homology model is unlikely to fully recapitulate the true human β-cardiac myosin IHM structure, and an actual high-resolution structure has been desperately needed. This structure has now been obtained and reported by Robert-Paganin et al. at the 2022 Gordon Research Conference (Cytoskeletal Motors) meeting. We will soon have the true structural information to properly assess the destabilizing effects of HCM mutations on the IHM off-state. But even with the true human β-cardiac myosin IHM structure, for such an allosteric molecule, it is difficult to predict in silico which mutations will increase N_a_. Functional tests described in this review using purified human β-cardiac myosin containing the ventricular human cardiac light chains are required to biochemically assess the effects of any particular HCM mutation (Table 1). Nonetheless, the mesa hypothesis (Spudich, 2015), which states that increasing N_a_ is a unifying hypothesis for the cause of hypercontractility by HCM mutations, now has a good deal of support experimentally.

The stability of the IHM is likely regulated by other proteins in the sarcomere, including MyBPC. Mutations in MyBPC are known to cause about one-third of genetically defined HCM cases, with many cases thought to be caused by haploinsufficiency due to frameshift, nonsense or splice site mutations that result in premature termination codons (Helms et al., 2020). This fits well with the mesa hypothesis, since a stabilizing effect of MyBP-C on the IHM state would be relieved by loss of some MyBP-C in the sarcomere. There are, however, also MyBP-C missense mutations that cause HCM and their mechanisms of action are not well defined (Harris et al., 2011). Decades of studies on MyBPC has revealed binding interactions with actin to activate the thin filament, and binding to myosin to control myosin head availability through the thick filament (Heling et al., 2020). Its ability to bind to both actin and myosin with similar affinities, and unique spatial localization within the sarcomere makes MyBPC a particularly interesting and challenging molecule to study. Titin is also an important player in sarcomere function and will likely have direct effects on thick filament activity by interacting with MyBP-C and either the HMM portion of the myosin or the LMM core of the thick filament which is involved in the sequestering of myosin heads (Spudich, 2019). It is important to note that, while titin mutations are the most commonly identified genetic etiology of DCM (∼30%), it is rarely associated with HCM (Herman et al., 2012; LeWinter and Granzier, 2013). The reason for this skewed phenotypic effect is unclear, and the function of titin requires further investigation.

The recent development of pharmacotherapy with myosin modulators sheds further light on the regulation of sarcomere function (Trivedi et al., 2018; Spudich, 2019; Nag et al., 2021). Increasing the ensemble force of the sarcomere can be achieved through three different mechanisms: *1*) thick filament activation that leads to more myosin heads released and available to bind to actin, *2*) enhanced thin filament activation that allows more myosin heads to bind to actin, which in turn cooperatively opens more myosin binding sites and promotes more crossbridge formation, and *3*) enhanced biochemical/biophysical properties of the myosin motor. It should be noted that these three mechanisms are not mutually exclusive, and in fact there are likely many crossover effects. For example, OM stabilizes the PPS state, which pulls more myosin out of the IHM state, but OM also causes slowing of the actin-induced rotation of the myosin light chain binding domain during the actin-myosin cycle, and this myosin state enters a prolonged actin-bound state that likely activates the RTF by moving tropomyosin away from the myosin binding site on actin (Rohde et al., 2017). Further drug development targeting sarcomere function will require attention to the impact on overall ensemble force from all three mechanisms.

The characterization of the primary effects of HCM mutations on human cardiac myosin has revealed many aspects of sarcomere function that can be altered by a single amino acid change. As discussed above, there is growing evidence that these HCM mutations cause hypercontractility which leads over time to hypertrophy. These effects have been recapitulated at the cellular level, demonstrating the usefulness of induced pluripotent stem cell-derived cardiac myocytes as a system for assessing the effect of mutations in either a patient-derived or isogenic background (Vander Roest et al., 2021). How the increased contractility is perceived by the cardiac myocyte is as yet unclear, and will be critical to understand the mechanism of cellular hypertrophy, myocyte disarray and the development of fibrosis. Increases in contractility could increase overall ATP utilization, resulting in energy imbalance (Abel and Doenst, 2011; Ashrafian et al., 2011), Ca^2+^ dysregulation and changes in wall tension that can trigger hypertrophic signaling (van Berlo et al., 2013; Davis et al., 2016). Therapeutic interventions to either reduce or augment myosin function hold promise for unmet clinical needs in cardiomyopathy and heart failure of various etiologies, respectively, as shown in the multiple clinical trials described above. Increasingly precise understanding of the alterations in contractility in disease states like HCM will continue to yield more sophisticated therapeutic interventions.
