# Erratum: Hypertrophic cardiomyopathy: Mutations to mechanisms to therapies

**DOI:** 10.3389/fphys.2022.1111059

**Published:** 2022-12-12

**Authors:** Frontiers Production Office

**Affiliations:** Frontiers Media SA, Lausanne, Switzerland

**Keywords:** myosin, hypertrophic cardiomyopathy, super relaxed state, mavacamten, omecamtiv mercarbil

Due to a production error, there was a mistake in Table 1 as published. The references listed in the table were accidentally inverted. The corrected Table 1 appears below.

**TABLE 1:** Summary of the functional effect of HCM mutations in cardiac myosin.

HCM mutation	Intrinsic force (F_intrinsic_)	Velocity (v)	ATPase (k_cat_)	Number of available myosin heads (N_a_)	References
Change from wildtype human β-cardiac myosin					
R403Q	↓	↑	↑	↑	Nag et al. (2015), Nag et al. (2017), Sarkar et al. (2020)
R453C	↑	↓	↓	-	Sommese et al. (2013) Nag et al. (2017), Bloemink et al. (2014)
R719W	↓	↑	NC	↑	Kawana et al. (2017), Adhikari et al. (2019)
R723G	↓	↑	NC	-	Kawana et al. (2017)
G741R	NC	NC	NC	-	Kawana et al. (2017)
R663H	NC	NC	NC	↑	Sarkar et al. (2020)
R249Q	-	↓	↓	↑	Nag et al. (2017), Adhikari et al. (2019)
I457T	-	↑	↑	NC	Adhikari et al. (2019)
P710R	- *	↓	↓	↑	Vander Roest et al. (2021), Vera et al. (2019)
V763M	-	↑	NC	-	Vera et al. (2019)
H251N	↑	↑	↑	↑	Adhikari et al. (2016), Nag et al. (2017), Adhikari et al. (2019), Vera et al. (2019)
D239N	↑	↑	↑	-	Adhikari et al. (2016)
D778V	↓**	↑	↑	↑	Morck et al. (2022)
L781P	NC**	↓	NC	↑	Morck et al. (2022)
S782N	↓**	NC	NC	↑	Morck et al. (2022)
A797T	-	NC	NC	↑	Morck et al. (2022)
F834L	-	NC	NC	↑	Morck et al. (2022)

Summary of HCM mutations that have been studied using the purified human cardiac myosin heavy chains and light chains. While F_intrinsic_, v and k_cat_ values showed no consistent trends among the HCM variants, the N_a_ was increased in all tested variants. * The intrinsic force has not been measured for this mutation. Optical trapping using harmonic force spectroscopy showed a reduction in the step size of the mutant myosin motor and decreased load sensitivity of the actin detachment rate at the single molecule level. ** The intrinsic force has not been measured for this mutation. Optical trapping using harmonic force spectroscopy was used to obtain load-dependent detachment rate, load sensitivity and step size. The average force of the sarcomere was calculated using these parameters.

Due to a production error, the **Reference** for “Vera et al., 2019” was incorrectly written as “Vera, C. D., Johnson, C. A., Walklate, J., Adhikari, A., Svicevic, M., Mijailovich, S.M., et al. (2019). Myosin motor domains carrying mutations implicated in early or late onset hypertrophic cardiomyopathy have similar properties. Biophysics. doi:10.1101/622738”. It should be “Vera, C. D., Johnson, C. A., Walklate, J., Adhikari, A., Svicevic, M., Mijailovich, S.M., et al. (2019). Myosin motor domains carrying mutations implicated in early or late onset hypertrophic cardiomyopathy have similar properties. Biophysics. doi:10.1074/jbc.RA119.010563”. The publisher apologizes for this mistake. The original version of this article has been updated.

